# Cross-Talk between P2X and NMDA Receptors

**DOI:** 10.3390/ijms21197187

**Published:** 2020-09-29

**Authors:** Larry Rodriguez, Catherine Yi, Cameron Chu, Quentin Duriez, Sharyse Watanabe, Megan Ryu, Brandon Reyes, Liana Asatryan, Eric Boué-Grabot, Daryl Davies

**Affiliations:** 1 Department of Pharmacology and Pharmaceutical Sciences, School of Pharmacy, University of Southern California, Los Angeles, CA 90033, USA; larryrod@usc.edu (L.R.); yich@usc.edu (C.Y.); chucamer@usc.edu (C.C.); sharysew@usc.edu (S.W.); meganryu@usc.edu (M.R.); branreyes2970@gmail.com (B.R.); 2 Univ. de Bordeaux, Institut des Maladies Neurodégénératives, UMR 5293, F-33000 Bordeaux, France; quentinduriez@protonmail.com (Q.D.); eric.boue-grabot@u-bordeaux.fr (E.B.-G.); 3 CNRS, Institut des Maladies Neurodégénératives, UMR 5293, F-33000 Bordeaux, France; 4 Titus Family Department of Clinical Pharmacy, School of Pharmacy, University of Southern California, Los Angeles, CA 90033, USA; asatryan@usc.edu

**Keywords:** NMDA receptors, P2X2 receptors, P2X4 receptors, cross-talk

## Abstract

Purinergic P2X receptors (P2X) are ATP-gated ion channels widely expressed in the CNS. While the direct contribution of P2X to synaptic transmission is uncertain, P2X reportedly affect N-methyl-D-aspartate receptor (NMDAR) activity, which has given rise to competing theories on the role of P2X in the modulation of synapses. However, P2X have also been shown to participate in receptor cross-talk: an interaction where one receptor (e.g., P2X2) directly influences the activity of another (e.g., nicotinic, 5-HT3 or GABA receptors). In this study, we tested for interactions between P2X2 or P2X4 and NMDARs. Using two-electrode voltage-clamp electrophysiology experiments in *Xenopus laevis* oocytes, we demonstrate that both P2X2 and P2X4 interact with NMDARs in an inhibited manner. When investigating the molecular domains responsible for this phenomenon, we found that the P2X2 c-terminus (CT) could interfere with both P2X2 and P2X4 interactions with NMDARs. We also report that 11 distal CT residues on the P2X4 facilitate the P2X4–NMDAR interaction, and that a peptide consisting of these P2X4 CT residues (11C) can disrupt the interaction between NMDARs and P2X2 or P2X4. Collectively, these results provide new evidence for the modulatory nature of P2X2 and P2X4, suggesting they might play a more nuanced role in the CNS.

## 1. Introduction

Ionotropic receptors are ligand-gated ion channels (LGICs) responsible for various physiological processes. These LGICs, widely expressed in neurons, are activated by specific chemical species, such as glutamate, adenosine triphosphate (ATP), or γ-aminobutyric acid (GABA), with multiple receptor families being found across a diverse population of cell types [1]. Glutamate receptors are one of the largest and most widely expressed families of excitatory LGICs found in the CNS. Three different classes of ionotropic glutamate receptors exist, differentiated by their ability to be stimulated by selective agonists: Kainate, α-amino-3-hydroxy-5-methyl-4-isoxazolepropionic acid (or AMPA), and N-methyl-D-aspartate (or NMDA) receptors. NMDA receptors are heterotetramers, usually consisting of two obligate GluN1 subunits, and either two GluN2 or two GluN3 subunits. Within the NMDA type of glutamate receptors, there exist several subtypes of GluN2 (i.e., GluN2A-D), each with a different cytoplasmic domain, resulting in differences in functional and physiological activities [2].

ATP is an important signaling molecule in the CNS, as it activates purinergic receptors, including the ATP-gated cation channel family (Purinergic P2X (P2X) receptors) which have been shown to play a role in neuroinflammation, pain, and neurological dysfunction. Among the members of the P2X family (consisting of P2X1–P2X7), P2X2, P2X4, and P2X6 subtypes are generally expressed on most neurons, and are regularly found at the edge of the post-synaptic densities of excitatory synapses [3]. P2X subtypes also show similar structural characteristics: an amino-terminal intracellular domain, two transmembrane domains, and a carboxy-terminal (CT) intracellular domain. In fact, the closed and open zebrafish P2X4 crystal structures (PDB: 4DW0 and 4DW1, respectively) have previously been used to build other P2X structural models, namely P2X2, highlighting the conserved shape of P2Xs [3].

While ATP is released by neurons (as well as by glial cells in the CNS), direct evidence supporting the function of P2X in synaptic transmission is limited. ATP can be coreleased with GABA or glutamate at the central synapse [4,5], suggesting a modulatory role in synaptic activity or plasticity in the brain. For example, studies on P2X4 knockout (P2X4 KO) mice suggested that calcium entry via P2X4 played a role in the induction of long-term potentiation (LTP) via modulation NMDARs [6]. Additional studies on P2X4 KO mice support the notion that P2X4 modulates NMDARs, although results indicated that calcium influx from P2X4 alone was not sufficient to explain changes in synaptic plasticity [7]. More recent studies reported that P2X can down-regulate NMDARs in a calcium-dependent manner [8], raising more questions regarding the mechanistic function of P2X4. Roles for P2X4 in behavior have continued to emerge; studies have found that P2X4 KO mice: (1) show cognitive-behavioral deficits, (2) consume significantly more ethanol as compared to wildtype controls and (3) display aberrant signaling within the mesolimbic pathway of the brain [9,10,11]. Moreover, pharmacologic and genetic studies support the significance of P2X4 in cognitive function (for a detailed review on P2X modulators in disease, see [12]); P2X4 positive allosteric modulators (e.g., ivermectin and moxidectin) have been shown to reduce ethanol intake in wildtype, [13,14] and internalization-deficient P2X4 knock-in mice, which display increased surface expression of P2X4, demonstrate that P2X4 regulates anxiety and memory processes [15]. Indeed, increased P2X4 surface expression in excitatory neurons was shown to alter long-term depression and long-term potentiation (LTD and LTP) in the hippocampus, consistent with the idea that post-synaptic P2X4 receptors may regulate NMDAR function. While these studies indicate that P2X are integral in neuronal signaling and cognitive disease states, determining how P2X mediate these effects is necessary for determining their promise as a target for cognitive pathologies (for a recent review of P2X4 in the nervous system, see [16]).

A large body of evidence suggests that a major function of P2X involves interacting with and regulating other LGICs (i.e., cross-talk). In cross-talk, coactivation of P2X and another receptor leads to rapid inhibition of agonist-evoked currents. P2X cross-talk can rely on physical interactions between the intracellular domains of the two different receptors and may also regulate the subcellular targeting of receptors in neurons. Cross-talk between several P2X subtypes has been shown to modulate the activity of GABA-A receptors [17,18,19,20], nicotinic ACh receptors [21] and 5-Hydroxytryptamine Receptor 3A (5-HT_3A_) [22,23,24]. Alternatively, P2X can also have slow but long-lasting modulatory effects on the function or surface trafficking of receptors; activation of post-synaptic P2X by ATP released from glia has been shown to trigger changes in the surface trafficking of α-amino-3-hydroxy-5- methyl-4-isoxazolepropionic acid receptors (AMPAR), which leads to long lasting changes in synaptic efficacy at glutamatergic synapses. In the hypothalamus, activation of P2X7 by ATP led to increases in the number of surface AMPAR and synaptic strength [25]. In the hippocampus, activation of post-synaptic P2X2 or P2X4 can activate surface AMPAR internalization, leading to a P2X-mediated long term synaptic depression [26,27]. Despite evidence of the modulatory potential of P2X, interactions between P2X and NMDAR have not been investigated.

To better understand how P2X regulate NMDAR function [6,7,28] here we investigated putative interactions between P2X and NMDAR using two-electrode voltage clamp (TEVC) electrophysiology in *Xenopus laevis* oocytes coexpressing P2X2 or P2X4 and various GluN2-containing NMDAR combinations. We demonstrate an interaction between P2X and NMDARs, producing inhibited responses, and that this interaction between both receptor types exhibits subunit-dependent properties. Using mutagenesis and molecular biology approaches, we delved deeper into the domains responsible for this interaction and found evidence suggesting that the C-terminal of P2X is important for the interactions between P2X and NMDARs. 

## 2. Results

### 2.1. Coactivation of P2X and NMDA Receptors

**Figure A1:**
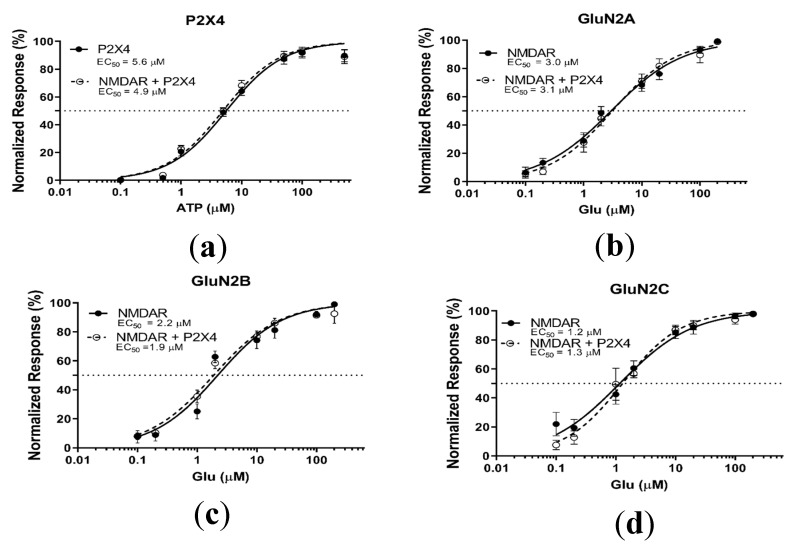
Comparison of the concentration-response curves for P2X4Rs or NMDARs expressed individually (depicted with solid lines) or together (depicted with dotted lines) in *Xenopus leavis* oocytes. (**a**) ATP concentration-response curves. EC_50_ values obtained from ATP-concentration curves of individual P2X4 and P2X4 coexpressed with NMDARs were not significantly different. (**b**–**d**) Glutamate-concentration response curves. EC_50_ values were not statistically significantly different for Glu-concentration response curves for individual GluN2A, GluN2B, GluN2C (solid lines) and each NMDAR subtype coexpressed with P2X4 (dotted lines). P2X4 and NMDARs were injected at respectively 20 ng and 10 ng cRNAs. Data represent Mean ± SEM. Statistical analysis performed using Exact sum-of-squares F-test. (**a**) *p* > 0.5; n = 9–12; (**b**) *p* > 0.5; n = 9–12; (**c**) *p* > 0.3; n = 9–12; (**d**) *p* > 0.5; n = 9–12.

**Figure A2:**
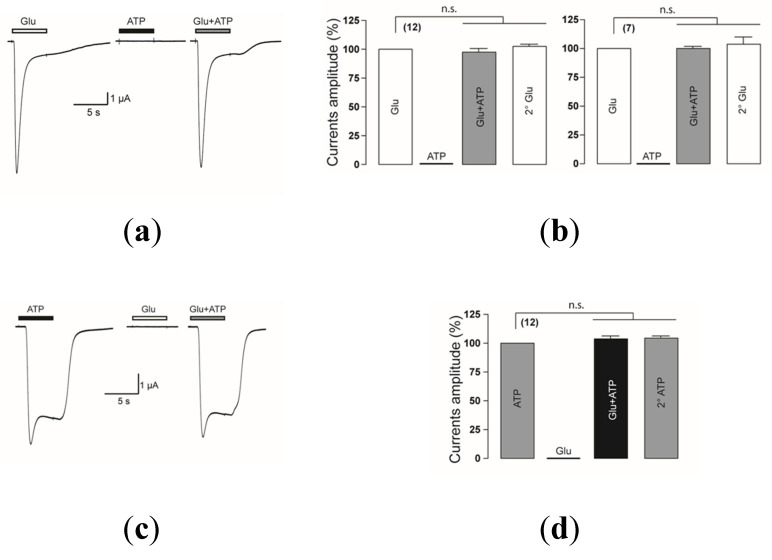
Application of ATP agonist does not affect Glu responses when NMDARs are expressed alone and conversely, application of Glu does not modulate ATP responses when P2X are expressed alone. (**a**) Representative current recorded from an individual oocyte GluN2B-containing NMDARs responding to 100 µM: Glu (left), ATP (middle), or Glu + ATP (right) are shown.; (**b**) Bar graphs representing the current obtained from application of agonists, normalized to the Glu response for each GluN2B-expressing oocyte.; (**c**) Representative current recorded from an individual oocyte expressing only P2X2 responding to 100 µM: ATP (left), Glu (middle), or Glu + ATP (right) are shown. (**d**) Bar graphs representing the current obtained from application of agonists, normalized to the ATP response for each P2X2-expressing oocyte. The data are expressed as mean ± SEM. *ns* > 0.05 (one-way ANOVA).

ATP and glutamate (Glu) are coreleased from presynaptic vesicles [4], suggesting that activation of post-synaptic P2X and NMDARs might occur at the same time. We expressed P2X and NMDARs separately or in combination in *Xenopus laevis* oocytes. Voltage clamp recordings demonstrate that coexpression did not affect either ATP or Glu concentration-responses when tested separately (Figure A1). Furthermore, ATP did not affect NMDAR responses when expressed alone, and Glu did not affect P2X responses when expressed alone (Figure A2). Note that since NMDARs are heterotetramers consisting of obligate GluN1 and variable GluN2 subunits, we will refer only to the variable GluN2 subunit when discussing differences among NMDARs. Furthermore, all applications containing Glu also contain a saturating concentration of glycine (10 µM).

We sought to characterize the effects of activating both receptor types at the same time (coactivation). If both receptors are functionally independent, then simultaneous activation of P2X and NMDAR should be additive. That is, equal to the sum of the separate response of each receptor when activated individually by their respective agonist [17,20,21,22]. On the other hand, non-additive responses during concomitant application of both agonists would indicate a functional interaction with synergistic (greater than additive responses) or inhibited (less than additive response) effects. 

#### 2.1.1. Coactivation of P2X4 and NMDA Receptors Produces Non-Additive (Inhibited) Responses

**Figure 1:**
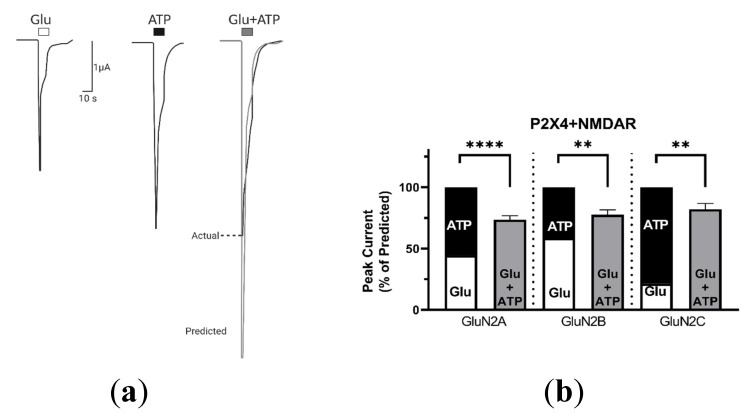
P2X4–NMDAR coactivation produces an inhibited response. (**a**) Representative currents recorded from an individual oocyte coexpressing P2X4 and GluN2B-containing NMDARs responding to: Glutamate (Glu, 2 µM), ATP (5 µM), or Glu and ATP (2 µM and 5 µM, respectively). The predicted additive response (grey line) is calculated as the sum of the separate Glu and ATP induced currents. (**b**) Bar graphs comparing the predicted and actual responses obtained from coapplication of agonists for P2X4 and NMDARs containing GluN2A (*n* = 9), GluN2B (*n* = 9), or GluN2C (*n* = 10), normalized to the sum of the separate Glu and ATP responses for each oocyte. The data are expressed as mean ± SEM; Statistical analysis performed using paired *t*-test ** *p* < 0.01, **** *p* < 0.0001. P2X: Purinergic P2X receptors; NMDAR: N-methyl-d-aspartate receptor.

**Figure 2:**
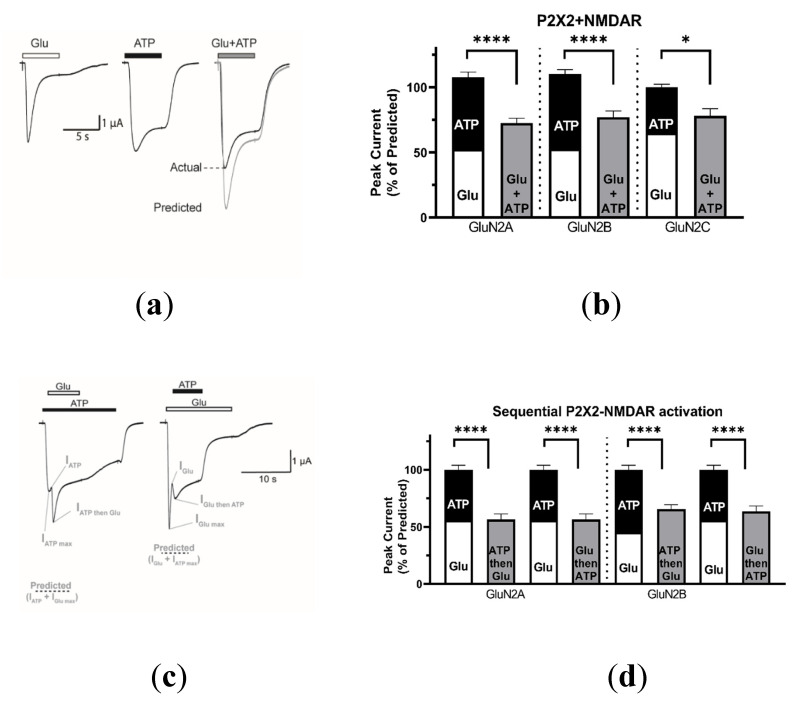
P2X2–NMDAR coactivation produces an inhibited response. (**a**) Representative current recorded from an individual oocyte coexpressing GluN2B-containing NMDARs and P2X2 responding to 100 µM: Glu (left), ATP (middle), or Glu + ATP (right) are shown. (**b**) Bar graphs comparing the predicted and actual responses obtained from coapplication of agonists for P2X2 and NMDARs containing GluN2A (*n* = 22), GluN2B (*n* = 21), or GluN2C (*n* = 6), normalized to the sum of the separate Glu and ATP responses for each oocyte. (**c**) Representative current from an individual oocyte coexpressing P2X2 and NMDARs containing GluN2B. For sequential activation of P2X2 and NMDARs, primary application of either ATP (left) or Glu (right) first appears to reduce subsequent coactivation responses. The predicted response when ATP is applied first is calculated as the sum of the current response to ATP immediately before Glu is coapplied and the maximum current response to Glu thereafter. This order is reversed when calculating the predicted response when Glu is applied first. (**d**) Bar graphs of P2X2 and GluN2A (*n* = 16) or GluN2B (*n* = 17) containing NMDARs, comparing the predicted and actual responses obtained from sequential activation and coapplication of agonists, normalized to the sum of the predicted current. The data are expressed as mean ± SEM; Statistical analysis performed using paired *t*-test * *p* < 0.05, **** *p* < 0.0001.

As presented in Figure 1a, coapplication of both agonists on oocytes coexpressing P2X4 and NMDARs consisting of GluN2B subunits produced a significantly lower current response (black line) than the arithmetic sum of the separate responses evoked by application of Glu and ATP alone (grey line). The bar graph presented in Figure 1b illustrates the mean of the predicted sum of the Glu responses (white) and ATP responses (black) and the mean of actual peak currents evoked during coactivation of P2X4 and different GluN2-containing NMDARs (grey), normalized to the predicted response (set as 100%). Regardless of the GluN2 subunit, we found that coactivation of P2X4 and NMDARs produced significantly smaller responses than predicted: GluN2A, GluN2B, and GluN2C produced 73.6 ± 3.1% (*p* = 0.000099; *n* = 9), 77.7 ± 3.9% (*p* = 0.00103; *n* = 9), and 82.2 ± 4.7% (*p* = 0.004; *n* = 10) of the predicted coactivation response, respectively. These results indicate that P2X4 and NMDARs do not function in isolation and that coactivation leads to inhibited responses, independent of the GluN2 subunit composition of NMDARs (one-way ANOVA, *p* > 0.05). Interestingly, we observed an increase in Glu responses after coactivation for GluN2A- and GluN2C-containing NMDARs (GluN2B was not assessed). Unfortunately, since these Glu responses remained higher than those obtained before for Glu+ATP coactivation (see Figure A6a), determining the directional nature of this interaction (i.e., does activation of P2X4 first reduce the coactivation response of NMDARs, or vice versa?) was not further investigated in this current work (for example see Figure 2c,d). 

#### 2.1.2. Coactivation of P2X2 and NMDA Receptors Produces Inhibited (Cross-Talk) Responses

Given that both P2X2 and P2X4 are widely expressed in the CNS, we wanted to determine if P2X2 could also interact with NMDARs. In a similar manner, we coexpressed P2X2 and NMDARs in oocytes and recorded the currents evoked by application of ATP (100 µM), Glu (100 µM) or both agonists (100 µM each) (Figure 2). Similar to P2X4, P2X2 appeared to interact with NMDARs, as coactivation of P2X2 and NMDARs (containing GluN2A, GluN2B, or GluN2C) produced significantly lower responses than predicted (72.5 ± 3.8%, *n* = 21, *p* = 0.0000022; 77.1 ± 4.8%, *n* = 22, *p* = 0.000024; 77.9 ± 5.6%, *n* = 6, *p* = 0.015, respectively). Unlike P2X4, NMDAR responses after P2X2 coactivation fully recovered (data not shown). Thus, to investigate the directional nature of this phenomenon, we added the agonists sequentially; i.e., ATP was coapplied when the Glu response reached its maximum or vice versa—Glu was coapplied when the ATP response reached its peak. As shown in Figure 2c, application of either ATP during Glu-evoked current or Glu during ATP-evoked current both led to responses that were significantly lower than the predicted sum of the individual responses. Furthermore, we report that current inhibition occurs at various holding potentials (ranging from −60 mV to 0 mV), indicating that cross-talk between P2X2 and NMDARs is voltage-independent (see Figure A4). Additionally, at these less depolarized potentials, reduced agonist responses are observed, yet inhibition persists, suggesting that inhibition is not due to current saturation. Collectively, these results suggest that P2X2 and NMDARs do not function in isolation and that an interaction leads to a functional and reciprocal cross-inhibition that is independent of the GluN2 subunits composition of the NMDARs. 

### 2.2. P2X4–NMDAR Interactions Are Independent of Ca2+ Influx

**Figure 3:**
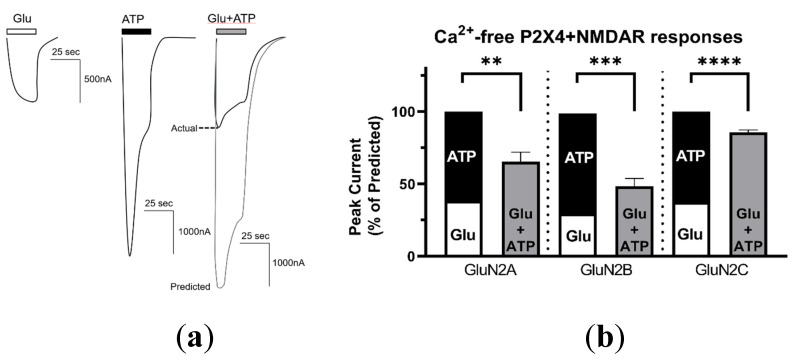
P2X4–NMDAR cross-talk is independent of calcium. (**a**) Representative currents recorded in Calcium-free Ringers’ solution (CfRS) from an individual oocyte coexpressing P2X4 and GluN2B-containing NMDARs responding to: Glu (2 µM), ATP (5 µM), or Glu and ATP (2 µM and 5 µM, respectively). The predicted additive response (grey line) is calculated as the sum of the individual Glu and ATP induced currents. (**b**) Bar graphs representing the predicted and actual responses obtained from coapplication of agonists, normalized to the sum of the separate Glu and ATP responses for each oocyte. For GluN2A, coactivation produced a statistically lower response than the predicted response (*p* < 0.01; paired *t*-test; *n* = 10). The same result was observed for GluN2B (*p* < 0.001; paired *t*-test; *n* = 9) and GluN2C (*p* < 0.0001; paired *t*-test; *n* = 28) coactivation. Furthermore, the degree of inhibition was not significantly different between the different GluN2 subunits (one-way ANOVA; *p* > 0.05) The data are expressed as mean ± SEM; statistical analysis performed using paired *t*-test, ** *p* < 0.01, *** *p* < 0.001, **** *p* < 0.0001.

P2X4 and NMDAR both display a high calcium permeability [29] which raises the question of whether the inhibited responses observed during P2X4–NMDAR coactivation could be mediated by or depend on calcium. At the same time, the increased responses to Glu after P2X4–NMDAR coactivation may be explained by calcium influx via P2X4 (see Figure A6a). Indeed, calcium influx has been shown to regulate NMDAR function, either by facilitating protein interactions [30] or activating downstream modulators [31,32]. To determine whether the putative P2X4–NMDAR interaction was mediated by calcium influx, we utilized a Calcium-free Ringer’s solution (CfRS) which substitutes barium chloride for the calcium chloride. In the absence of calcium, coactivation of P2X4 and NMDARs consistently produced significantly lower responses than we predicted: P2X4 and GluN2A, GluN2B, and GluN2C produced 65.5 ±6.5% (*p* = 0.0053), 48.2 ±5.4% (*p* = 0.00018), and 85.7 ± 6.2% (*p* = 0.00000003) of the predicted additive responses, respectively (Figure 3a). Additionally, the degree of inhibition was similar to if not greater than the inhibition obtained in Ca^2+^-containing medium (Figure 1) indicating that the Ca^2+^-influx through the opened receptor-channels does not mediate the observed inhibited interaction between P2X4 and NMDARs. Furthermore, in the absence of calcium, we observed that the Glu responses by NMDARs containing GluN2B remained lower after coactivation with P2X4 (Figure A6b), again preventing the determination of the directional nature of this interaction.

### 2.3. P2X4-Induced a Long-Lasting Inhibition of NMDAR Is GluN2-Subunit Dependent

**Figure 4:**
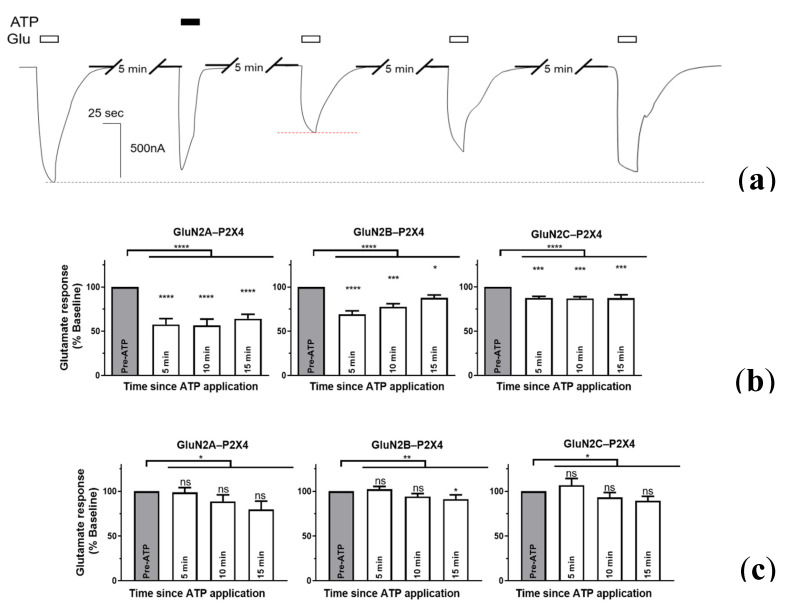
The time-course for recovery of P2X4-mediated NMDAR inhibition in the absence or presence of calcium. (**a**) Representative current evoked by application of Glu (2 µM), before and after activation of P2X4 by ATP (5 µM), from an individual oocyte coexpressing GluN2B-containing NMDARs and P2X4 in the absence of calcium (CfRS). Bar graphs representing the mean of the amplitude of NMDARs responses to Glu before and after P2X4 activation by ATP, either in the absence (**b**) or presence (**c**) of calcium. All values were normalized to the Glu response obtained before P2X4 stimulation. Glu responses after P2X4 activation were significantly lower NMDARs containing each of the GluN2A-C subunits in the absence of calcium (* *p* < 0.05, ** *p* < 0.01, *** *p* < 0.001, **** *p* < 0.0001, one-way ANOVA with Tukey’s post-hoc test; *n* = 10–17 oocytes). Additionally, the time course of glutamate current recovery appears distinct, i.e., GluN2-subunit specific. The data are expressed as mean ± SEM.

Activating P2X4 and NMDARs simultaneously (Figure 1) cannot inform the directionality of this interaction, and the effects of coactivation seem persistent (Figure A6), precluding sequential coactivation. To further investigate the duration of P2X4-induced inhibition of NMDAR, we first obtained stable NMDAR currents by applying Glu every 5 min and recorded, after a single P2X4 mediated current evoked by application of ATP, Glu-evoked responses over the time, in the presence or absence of calcium (Figure 4). We reasoned that, given the stability of Glu responses of NMDARs (see Figure A2b), changes to Glu responses after ATP application would indicate a sustained P2X4 interaction. As shown in Figure 4a, the amplitude of GluN2B response in CfRS recorded 5 min after P2X4 activation was significantly lower than the one recorded before P2X4 activation, representing ~70% of the baseline response and seemed to recover only partially over time (Figure 4b). GluN2A and GluN2B showed similar reduced responses to Glu 5 min after P2X4 activation; 57.8 ± 6.6% (*p* = 0.000046) and 69.1 ± 4.04% (*p* = 0.000028) of baseline, respectively. Consistent with the current inhibition observed during coapplication of both agonists (see Figure 1 and Figure 3), NMDARs containing GluN2C showed the lowest effect-size 5 min after P2X4 activation with 87.5 ± 1.9% of the baseline response to Glu (*p* = 0.00047). Recovery of the Glu responses over time was distinct among the different GluN2 subunits. NMDARs containing GluN2A do not recover 15 min after P2X4 activation, with Glu-induced current representing 64.3 ± 5.0% of the baseline responses to Glu. Similarly, GluN2C containing NMDARs, which showed the smallest effect size, behaved similarly after 15 min initial P2X4 activation, producing 87.3 ± 3.8% of the Baseline responses to Glu. On the other hand, NMDARs containing GluN2B seemed to recover more rapidly, although 15 min after P2X4 activation, responses to Glu remained significantly inhibited (*p* = 0.018) representing 87.9 ± 3.1% of the baseline response. Additionally, Glu responses 5 and 15 min after ATP were significantly different only for NMDARs containing GluN2B (*p* < 0.01), while the Glu responses for GluN2A and GluN2C remained similar while ATP remained the same (*p* > 0.05). Interestingly, while the presence of calcium (Figure 4c) produced significant differences between the NMDAR responses before and after P2X4 activation (*p* < 0.05), post-hoc analyses of Glu responses by GluN2A-C containing NMDARs were generally not significantly different from baseline (*p* > 0.05). Indeed, only GluN2B containing NMDARs showed a significant decrease in Glu responses, after 15 min. These results suggest that activation of P2X4 alone can induce a long-lasting inhibited response from NMDARs, the extent and duration of which depends upon the nature of GluN2 subunit. These results also suggest that, while P2X4–NMDAR inhibited interactions are independent of calcium influx, calcium entry via P2X4 can affect NMDAR function via a distinct mechanism.

### 2.4. Intracellular P2X Domains Mediate NMDAR Interactions

Inhibited forms of cross-talk were previously reported between several P2X subtypes and distinct members of the cys-loop receptor family (such as nicotinic receptors, GABA_A_ or 5-HT3 receptors) which led to reciprocal or unilateral inhibition observed only during the coactivation of both receptors. These previous cross-talk investigations suggest (by mutagenesis, peptide competition, or domain overexpression experiments) that the phenomena rely on physical interactions between motifs within the C-terminal tail (CT) of P2X subunits and the intracellular loop between TM3 and TM4 of cys-loop receptors [17,18,19,20,21,22,23]. Since our results indicated that P2X2 and P2X4 could interact with NMDARs in a similar manner, we investigated whether the CT of these subunits is required for the interaction with NMDAR and whether a motif shared by both P2X subunits confer the ability to interact with NMDARs. 

#### 2.4.1. P2X-NMDAR Inhibited Interactions Depend on C-Terminal P2X Domains

**Figure 5:**
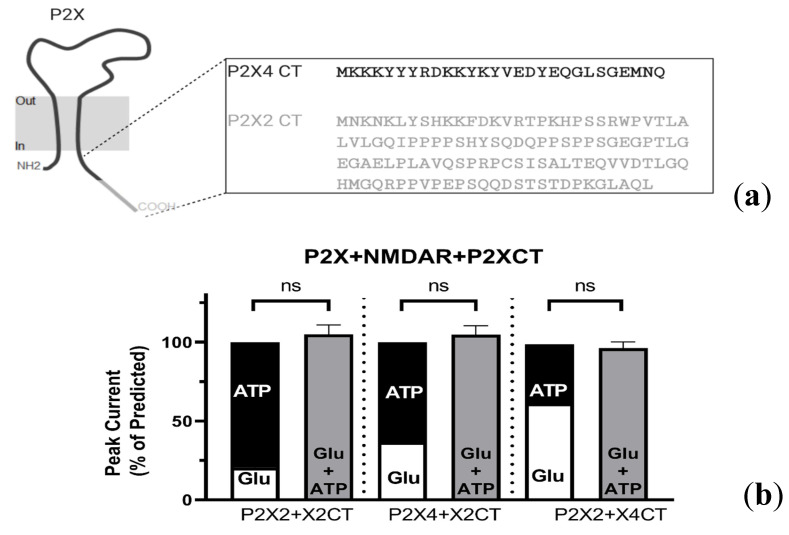
P2X c-terminus (CT) mediate interactions with NMDARs. (**a**) Top: A representative illustration of a homotrimeric P2X. The insert illustrates the differences in the size of the P2X4 (black) and P2X2 (grey) CT. (**b**) Bar graphs representing the predicted and actual responses obtained from coapplication of Glu (2 µM) and ATP (5 µM) in oocytes expressing either the P2X2 CT or the P2X4 CT, in combination with P2X2s or P2X4s and NMDARs. Agonist responses were normalized to the sum of the individual Glu and ATP responses for each oocyte. There was no statistically significant difference between the predicted responses and the actual responses produced by GluN2A-containing NMDARs and P2X4s in the presence of the P2X2 CT (105.0 ± 5.5%, *p* > 0.05, *n* = 7) Similarly, there was no statistically significant difference between the predicted responses and the actual responses produced GluN2A-containing NMDARs and P2X2s in the presence of the P2X4 CT (96.2 ± 3.9%, *p* > 0.05, *n* = 10) or the P2X2 CT (106.0 ± 6.21%, *p* > 0.05, *n* = 2). The data are expressed as mean ± SEM. Statistical analysis performed using paired *t*-test.

We reasoned that if the mechanism of NMDAR inhibition relies on residues located in either of the P2X CT (see Figure 5a), then coexpressing the CT domain of P2X4 (K373-Q388; P2X4 CT) with P2X2 and NMDAR would interfere and preclude inhibited cross-talk between P2X2 and NMDAR. Reciprocally, expression of the CT domain of P2X2 (M3374-L472; P2X2 CT) should interfere with P2X4–NMDAR interactions and alter the previously inhibited responses. As presented in Figure 5b, we found that the expression of a small construct (or minigene) encoding for only the P2X2 CT [17] in oocytes already coexpressing P2X4 and GluN2A-containing NMDARs was capable of interfering with the GluN2A-P2X4 interaction, as the current inhibition observed between P2X4 and GluN2A (see Figure 1) during the coapplication of both agonists was abolished (104.9 ± 5.5% of the prediction, *p* = 0.5164). Conversely, expression of a construct coding for P2X4 CT in oocytes already coexpressing GluN2A containing NMDARs and P2X2 was able to abolish the functional cross-inhibition interaction, as the coactivation response (96.2 ± 3.9%) was not significantly different from the predicted sum of the individual responses (*p* = 0.2944). As a positive control, the expression of the P2X2 CT in oocytes already coexpressing P2X2 and GluN2A-containing NMDARs abolished the cross-inhibition observed during P2X2-GluN2A coactivation (105.5 ± 6.2% of the prediction, *p* = 0.5949). It is unlikely that the inclusion of a CT domain reduced P2X expression (and therefore precluded inhibition), as the average peak P2X4 current when coexpressed with NMDARs was 2.94 ± 0.99 µA, while P2X4 coexpressed with NMDARs and the P2X2 CT was 5.62 ± 1.1 µA, which were not significantly different (*p* = 0.1362; unpaired *t*-test). These results suggest that neither P2X2 nor P2X4 interacted functionally with NMDAR in the presence of the CT domain of P2X2. These results also illustrate how cross-talk between P2X and NMDARs relies on the CT domain of P2X subunits. Overall, this work suggests that a common motif within the CT tail of P2X subunits can confer the ability to interact with NMDARs. To identify such motifs, we decided to perform a mutagenesis analysis on the CT of P2X4, which is shorter (in amino acid length) than P2X2 subunits.

#### 2.4.2. Resolving the P2X4 CT Domain Responsible for NMDAR Inhibition

**Figure 6:**
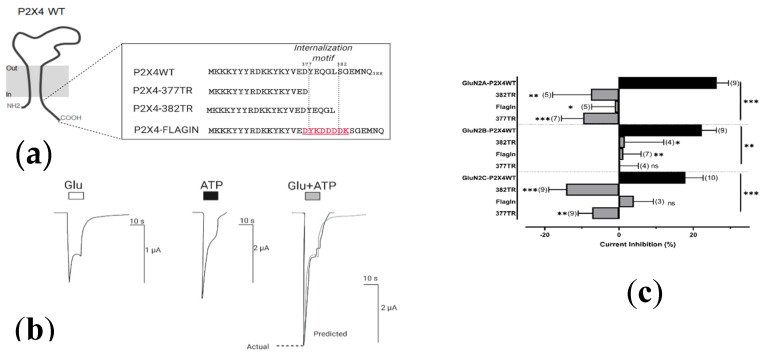
Residues in the P2X4 CT confer the ability to interact with NMDARs. (**a**) An illustration of the mutations performed on the P2X4 internalization motif, compared to the wildtype P2X4 (P2X4WT); (**b**) Representative currents recorded in Ringers’ solution from an individual oocyte coexpressing P2X4-377TR and GluN2A-containing NMDARs responding to: Glu (2 µM), ATP (1 µM), or Glu and ATP (2 µM and 1 µM, respectively). The predicted additive response (grey line) is calculated as the sum of the individual Glu and ATP induced currents. (**c**) Bar graphs representing the current inhibition obtained from coapplication of Glu and ATP for oocytes coexpressing different P2X4 mutants and NMDARs. Agonist responses were normalized to the sum of the individual Glu and ATP responses for each oocyte and subtracted from 100%. The data are expressed as mean ± SEM. P2X4 CT mutations were statistically significantly different (*p* < 0.05) from the previously obtained inhibited coactivation responses for each GluN2 subunit (Kruskal–Wallis test with Dunn’s post-hoc analysis). *ns* > 0.05, * *p* < 0.05, ** *p* < 0.01, *** *p* < 0.001. Parentheses denote number of replicates.

**Figure A3:**
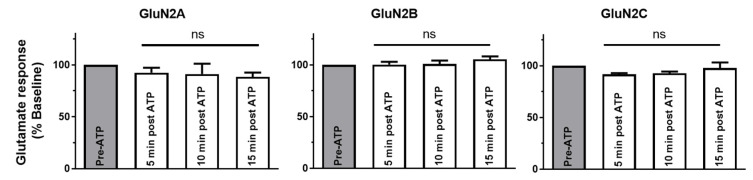
P2X4-mediated NMDAR inhibition depends on the P2X4 CT domain. Bar graphs representing the mean of the amplitude of NMDARs responses in CfRS before and after P2X4-377TR activation by ATP. All values were normalized to the Glu response obtained before P2X4-377TR stimulation. None of the Glu responses after ATP application were statistically significantly different (*p* > 0.05) from the baseline response (before ATP application) for any GluN2 subunit. The data are expressed as mean ± SEM. Statistical analysis performed using a one-way ANOVA test.

**Figure A4:**
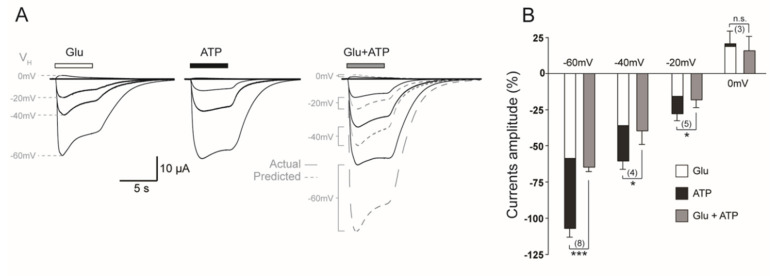
P2X2–NMDAR inhibition is voltage-independent. (**A**) Representative currents recorded at different holding potentials (V_H_) from an individual oocyte coexpressing NMDARs and P2X2 responding to 100 µM: Glu (left), ATP (middle), or Glu + ATP (right) are shown. (**B**) Bar graphs comparing the predicted and actual responses obtained at different holding potentials from coapplication of agonists for P2X2 and NMDARs, normalized to the sum of the separate Glu and ATP responses at each potential for each oocyte. The data are expressed as mean ± SEM; Statistical analysis performed using paired *t*-test * *p* < 0.05, *** *p* < 0.001. Parentheses denote number of replicates.

Among the seven P2X subunits, P2X4 is the only subunit to rely on a non-canonical motif (Y_378_XXGL_382_) of endocytosis [33,34] to undergo constitutive internalization. It is important to note that previously, GABA–P2X4 cross-talk was shown to be independent of this domain, relying instead on two other CT residues: Y_374_ and V_375_ [20]. To investigate whether the residues in the P2X4 CT that are responsible for P2X4 internalization are also responsible for the interaction with NMDARs, we truncated or replaced the P2X4 internalization motif, as illustrated in Figure 6a. We hypothesized that, if residues in the internalization domain of P2X4 are responsible for mediating NMDAR inhibition, then truncating the P2X4 receptor at residue 377 (P2X4-377TR) or replacing the internalization domain (YEQGL) of the wildtype P2X4 receptor with a FLAG epitope (DYKDDDK; P2X4-FlagIN) would abolish the inhibited effects of receptor costimulation. Similarly, if only the internalization motif were driving the interaction with NMDARs, then truncating P2X4 after the internalization motif, corresponding to residue 382, (P2X4-382TR) would still show inhibited coactivation responses. Figure 6b shows that the inhibited responses previously shown (i.e., Figure 2) were no longer present when coactivating either P2X4-377TR or P2X4-FlagIN. Furthermore, Figure A3 shows that P2X4-377TR failed to produce the long-lasting inhibition previously seen by full-length P2X4 in the absence of calcium, as shown in Figure 4b. Unexpectedly, we did not see any inhibited P2X4–NMDAR coactivation responses when P2X4s were truncated at residue 382, (P2X4-382TR) despite the inclusion of the P2X4 internalization motif in these mutant receptors. Collectively, these results suggest that the distal part of the CT tail, corresponding to the last 11 amino acids of P2X4, is necessary for P2X4 to functionally interact with NMDARs.

#### 2.4.3. Resolving the P2X4 CT Domain Responsible for NMDAR Inhibition

**Figure 7:**
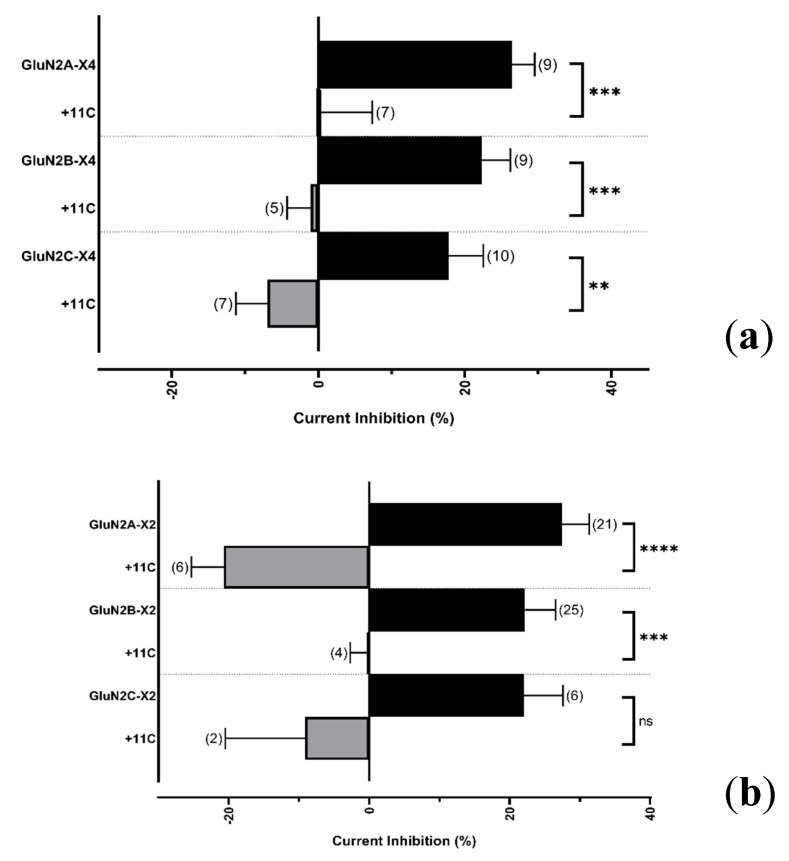
11C peptide disrupts P2X–NMDAR cross-talk. Bar graphs representing the current inhibition obtained from coapplication of Glu and ATP for oocytes expressing either P2X4 (**a**) or P2X2 (**b**) and NMDARs containing GluN2A-C, 30 min after injection with 11C (grey). The inhibited responses (black) are ablated by 11C. Agonist responses were normalized to the sum of the individual Glu and ATP responses for each oocyte and subtracted from 100%. The data are expressed as mean ± SEM and were analyzed using a Welch’s *t*-test. *ns p* > 0.05, ** *p* < 0.01, *** *p* < 0.001, **** *p* < 0.0001 Parentheses denote number of replicates.

Recombinant studies have shown that a peptide corresponding to the last 11 amino acids of P2X4 subunit (namely 11C) blocks P2X4 internalization and was previously used to determine the function of increased surface P2X4 in neurons from hypothalamus brain slices [20]. To confirm whether the distal domain of P2X4 is necessary and sufficient to ablate inhibited cross-talk with NMDARs, we reproduced the interaction–competition experiments as described in Figure 5, but instead of coexpressing the P2X4 CT, we injected the 11C peptide into oocytes (150 µM final concentration) expressing P2X2 or P2X4 in combination with NMDARs containing GluN2A, GluN2B, or GluN2C. Figure 7 shows that the presence of peptide 11C abolished the observed inhibited responses during the coactivation of both receptor types (see Figure 2b,d).

## 3. Discussion

### 3.1. P2X Modulation of NMDA Receptors

Our results are the first to provide direct evidence for and characterize P2X–NMDA interactions. We found that, when heterologously expressed in *Xenopus laevis* oocytes, P2X4 can interact with and inhibit NMDAR function consisting of GluN2A-C subunits (Figure 1). Furthermore, we report a similar inhibited phenomenon between P2X2 and NMDA receptors (Figure 2). These results indicate that P2X modulation of NMDARs may be more complicated and robust than the early reports [7,8,35]. 

**Figure A6:**
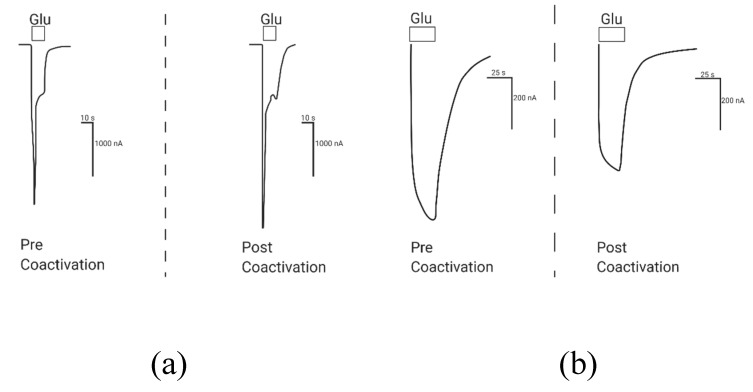
Altered response to Glu after P2X4–NMDAR coactivation. Representative current recorded from individual oocytes expressing P2X4 and NMDARs, responding to 2 µM Glu before (left) and after (right) Glu + ATP coactivation, in the presence (**a**) or absence (**b**) of calcium.

Our results support the hypothesis that P2X serves an important role in modulating the function of NMDARs and providing a new context for which to interpret the function of P2Xs; this has remained elusive, if not controversial. Early studies reported that P2X could contribute to synaptic transmission, albeit sparsely, and suggested that P2X could function as a “low-frequency filter”, suppressing NMDAR-mediated LTP under weak stimuli [35]. With the development of P2X4 KO mice came more support for a role for P2Xs in synaptic plasticity: (1) in the absence of P2X4, hippocampal neurons exhibited reduced LTP facilitation, and (2) Ivermectin, a P2X4 positive allosteric modulator, could increase LTP in wildtype mice, but not P2X4 KO mice [6]. These results suggested that P2X in the post-synaptic membrane modulate NMDAR function and LTP induction via calcium influx, rather than through synaptic transmission. However, studies have also demonstrated that P2X4 themselves must contribute to NMDAR modulation at post-synaptic densities, as intracellular administration of a calcium chelator could block NMDAR facilitation in WT mice, but had no effect in P2X4 KO mice. [7]. Indeed, our results indicate that these hypotheses regarding the mechanism of P2X modulation of NMDARs are not mutually exclusive, as we found that Calcium-free Ringer’s solution did not preclude P2X4–NMDAR cross-talk during receptor coactivation (Figure 3A) but that, in the presence of calcium, Glu responses by NMDARs after P2X4–NMDAR coactivation increase (Figure A6). It is important to note that oocytes express endogenous calcium-activated chloride channels. These channels have the potential to induce intracellular signaling cascades and would then contribute to a larger glutamate response being observed after coactivation in the presence of calcium, but not barium. Additionally, the order of coapplication was not assessed (e.g., Glu+ATP, then Glu, then ATP) which would indicate a rundown error if a difference were found. Despite these limitations, collectively, our results suggest that, while the interaction between P2X4 and NMDARs may be long-lasting, calcium influx via P2X4 plays a distinct role in modulating NMDARs. 

Until recently, the modulatory nature of P2Xs (including regulation of NMDARs) was inextricably linked to their ability to permeate calcium. However, with the characterization of a novel knock-in mouse strain, where P2X4 is fluorescently labeled and internalization-deficient (P2X4mCherryIn), a more nuanced role for P2X has emerged [15]. The study demonstrated that in CA1 synapses, P2X4mCherryIn mice displayed no changes in basal excitatory transmission, but exhibit changes to LTP and LTD induction. Considering that, in CA1 hippocampus neurons, LTP and LTD have been shown to rely on post-synaptic NMDARs [36] (for review on LTP see [37]), these results suggest that increased P2X4 activity in CA1 neurons alters NMDAR function, supporting the idea that P2X4s are involved in regulating synaptic plasticity.

### 3.2. P2X Intracellular Domains Mediate NMDAR Cross-Talk

Interactions of P2X2 and P2X4 were demonstrated for nicotinic [21], GABA [18], AMPA [27], and 5-HT_3A_ receptors [22,23]. This P2X-mediated regulation of other LGICs has been found to rely on diverse mechanisms: (1) physical protein-protein interactions, (2) receptor cotrafficking, or (3) signaling cascades, which can all be linked to direct interactions involving the receptor CT. P2X2 are similar in sequence and structure to P2X4, with the most prominent differences being found between the intracellular CTs. In an effort to better understand the receptor domains that drive this interaction, we chose to focus on the P2X4 CT, which is significantly shorter and thus, more simple to mutate [33,38]. Our studies show that truncation of P2X4 before the start of their internalization motif ablated NMDAR cross-talk, which leads one to believe that this interaction might be dependent upon P2X4 trafficking. Replacing only the internalization motif (YEQGL) with a flag epitope (DYKDDDKD) also ablated cross-talk, which seemed to confirm the significance of the domain. However, the P2X4 internalization motif does not seem to mediate this interaction entirely, as P2X4 truncated after the internalization motif also fails to interact with NMDARs (Figure 6). In support of this notion, the well characterized non-canonical tyrosine-based sorting motif *YXXGL* [33,34], was not shown to be responsible for the cross-talk interaction between P2X4 and GABA receptors [20].

While this resolves the interaction between P2X4 and NMDARs, does P2X2 interact with NMDARs via a similar mechanism? Based on the P2X4 truncation results, we reasoned that, if the P2X2 and P2X4 CT did mediate interactions with NMDA receptors, coexpressing the P2X4 CT in oocytes would interfere with P2X2–NMDAR cross-talk, and vice versa. As shown in Figure 5, our results are consistent with the hypothesis that each CT disrupted the inhibited responses that we presented in Figure 1 and Figure 2. Our mutation investigations suggested that the distal 11 CT residues of P2X4 seem to be required for NMDAR interaction, allowing us to hypothesize that suppression of cross-talk could be achieved by a peptide mimicking this domain. We synthesized and injected 11C as reported [20], and found that 11C ablated P2X2 and P2X4 interactions with NMDARs, suggesting that this interaction relies on several key residues found on the intracellular domains of P2X. Given that P2X2–NMDAR cross-talk was suppressed by 11C, our results also suggest that P2X2–NMDAR interactions rely on similar residues. 

How could the CT of both P2X4 and P2X2 mediate inhibition of NMDA receptors, given the size difference of these domains? Studies on the P2X4 CT and its non-canonical internalization motif revealed that, when co-crystallized with the µ2 adaptor protein (AP) subunit, residues 374 to 380 do not adopt a rigid structure [34]. Furthermore, the non-canonical YXXGL motif functions at the same canonical YXXΦ site due to the flexibility imparted by the glycine residue. When looking at the composition of the P2X2 CT, proline and other hydrophobic residues are prevalent. This is notable because proline can disrupt secondary protein structures and limit flexibility, while hydrophobic residues can promote a more “buried” conformation. It is possible that these residues allow the P2X2 CT to adopt a conformation that favors an interaction with NMDA receptors, much like in the case with P2X4. Unfortunately, no structural information exists for the P2X2 CT, which would provide more insight into their function. Despite these limitations, future studies can investigate the GluN2 region responsible for this P2X interaction, as well as the exact residues that mediate P2X–NMDAR cross-talk, using a point-mutation approach. Our results also raise the question of the extent to which ligand binding influences inhibition. P2X2–NMDAR interactions were observed at saturating concentrations of Glu and ATP, and P2X4–NMDAR interactions were observed at concentrations closer to EC_50_. While it would be interesting to see the extent of P2X–NMDAR inhibition at lower concentrations, such as EC_10-20_., such studies are out of the scope of this current work. However, it is important to note that non-additive interactions between P2X2 and 5-HT_3A_ have been, reported at both low and saturating agonist concentrations [22,23,24].

### 3.3. Resolving the Function of P2X in the Brain

Distinct forms of P2X cross-talk might serve discrete regulatory functions and arise from P2X mobility and localization, which has been shown to be subunit dependent. For example, P2X2 are highly mobile and stable at the cell surface, but rarely found on synaptic densities [39,40]. On the other hand, P2X4 are primarily found within intracellular compartments (due to constitutive internalization). Evidence has already shown that P2X4 play a role in integrating ATP signaling from astrocytes in the tripartite synapse, specifically by inhibiting GluN2B-containing NMDARs, an interaction that involves a multiprotein complex [8]. As such, P2X2 at extra synaptic densities may serve as a molecular “trap”, inhibiting NMDAs via an interaction that prevents their inclusion into the post-synaptic densities. The directional nature of this interaction might act as a negative feedback loop and allow for more diverse responses or fine-tuning. In contrast, P2X4s can act in a more targeted manner, waiting inside the cell and mobilizing into the post-synaptic density when stimulated. 

## 4. Materials and Methods 

### 4.1. Molecular Biology

Rat GluN receptor subunits were a kind gift from Dr. John Woodward. P2X4 cDNA was a kind gift from Dr. Iain Chessell and GlaxoSmithKline, and cloned into the pCDNA3.1 vector as previously described [41] while P2X2 cDNA was cloned into pCDNA3 [17]). pUNIV backbone was a gift from Cynthia Czajkowski (Addgene plasmid # 24705; http://n2t.net/addgene:24705; RRID:Addgene_24705) and was modified for subcloning of rat GluN subunits (to enhance RNA expression). Mutant receptors (P2X4 377-TR, P2X4-FlagIn) or P2X-CT minigenes were either available from previous studies [17,18,19,20,38] or mutated using the SuperFi PCR kit (ThermoFisher Scientific, Waltham, MA, USA) and transformed into Zymo Mix and Go competent cells (Zymo; Irvine, CA, USA). Single colonies were inoculated into Luria Broth and after 16–20 h, minipreps were performed using the ZymoPure miniprep kit. Plasmids were then restriction-digested with NotI-HF (New England Biolabs, Ipswich, MA, USA) and purified using the Zymo DNA clean-up kit. All constructs were sequence verified via sanger sequencing (Genewiz; La Jolla, CA, USA). The 11C peptide was synthesized by GenScript (Piscataway, NJ, USA), reconstituted (110 mM) in ultrapure water, and diluted to 10 mM using HEPES (10 mM; pH 7.2). Single-use aliquots were stored in −20 °C prior to injection. 

### 4.2. Xenopus Laevis Oocyte Injection and Electrophysiology

cRNA for experiments were synthesized with the Ambion message machine T7 kit (ThermoFisher Scientific, Waltham, MA, USA), purified using the Ambion MegaClear kit (ThermoFisher Scientific), and injected into *Xenopus laevis* oocytes (Ecocyte Biosciences, Austin, TX, USA). Previous studies have reported functional receptors using an injection concentration of approximately 10 ng of total NMDA RNA (5 ng of GluN1 and 5 ng of GluN2 subunit), 20 pg of P2X2 RNA, or 20 ng of P2X4 receptor RNA. As such, 20 ng P2X4 RNA or 10 ng of NMDAR RNA was injected alone or combined, with a final injection volume of 40 nL. For P2X2 studies, 20 pg of P2X2 RNA or 1 ng of total NMDAR RNA was injected alone or combined and injected into each cell, with a final injection volume of 40 nL. Aliquots of RNA were prepared, diluted with RNAsecure solution (ThermoFisher Scientific), and stored at −80 °C until use, to minimize RNAse degradation. 11C peptide injections were performed 30 min before two-electrode voltage clamp recordings. 15 nL of 10 mM 11C peptide in 4-(2-hydroxyethyl)-1-piperazineethanesulfonic acid (HEPES), was injected into each oocyte, for an approximate intracellular concentration of 150 µM. 1ng of CT minigenes were injected into oocytes in an injection volume of 20 nL, with injections performed 1 day before recordings. All injections were performed using a NanoJect III system (Drummond Scientific; Broomall, PA, USA). Recordings were performed 1–3 days after cRNA injection. Two-electrode voltage clamp recordings were performed using previously established methods [41,42]. In brief, oocytes were placed in the RC-3Z oocyte recording chamber (27 × 3.2 × 3.2 mm bath volume configuration) and membrane potentials were clamped at −70 mV using the OC-725C oocyte clamp (Warner Instruments, Hamden, CT, USA), and the oocyte recording chamber was continuously perfused with Ringer’s solution ± agonist using a Dynamax peristaltic pump (Rainin Instrument Co., Emeryville, CA, USA) at 3 mL/min using an 18-gauge polyethylene tube (Becton Dickinson, Sparks, MD, USA). All perfusion solutions contain a buffer solution consisting of 115 mM NaCl, 2.5 mM KCl, 1.8 mM CaCl_2_ (or 1.8 mM BaCl_2_ to avoid calcium-induced current generated by Ringer’s solution), and 10mM HEPES, with a final pH of 7.2. A 10 µM glycine stock was prepared using perfusion solution, which was used to dissolve and prepare solutions containing glutamate. ATP solutions were prepared fresh daily using stocks of 10 mM, which were dissolved in perfusion solution and stored at −20 °C. In the presence of calcium, Glutamate and/or ATP were applied for 10 s (to reach a peak current response). In the absence of calcium, (i.e., Calcium-free Ringer’s solution or CfRS) Glutamate and/or ATP were applied for 25 s. A wait time of at least 5 min of perfusion buffer occurred between any agonist applications to ensure complete washout of agonist. The resulting currents were filtered at 5 kHz and recorded using an analog chart recorder (P2X4 studies) or digitized using a Digidata 1320 (P2X2 studies). The voltage-clamp was continuously monitored for errors (i.e., failure to maintain −70 mV), in which case data were not used. All current values obtained were normalized to the most recent stable responses obtained immediately before agonist coapplications began, unless stated otherwise. Figures were created with BioRender.com.

### 4.3. Data Analysis 

Data were obtained from several batches of oocytes from at least three different frogs, and are expressed as mean ± S.E.M. The effects of costimulation are presented as percentages, normalized to the sum of the stable currents evoked by ATP and glutamate alone on each individual oocyte. The percentage of the inhibition is presented as the costimulation response subtracted from 100%. The Prism 8 software suite (GraphPad Software, Inc., San Diego, CA, USA) was used for data analysis and curve fitting. Statistical analysis was performed by log-transforming costimulation responses, then using Student’s paired *t*-test or one-way ANOVA followed by a Bonferroni post-hoc comparison. Log transformation of percent inhibition is not possible (as negative values are observed) so these data were analyzed using a Kruskal–Wallis test with a Dunn’s test for multiple comparisons or Welch’s *t*-test, as noted. Significance was set at *p* < 0.05.

## Figures and Tables

**Figure A5:**
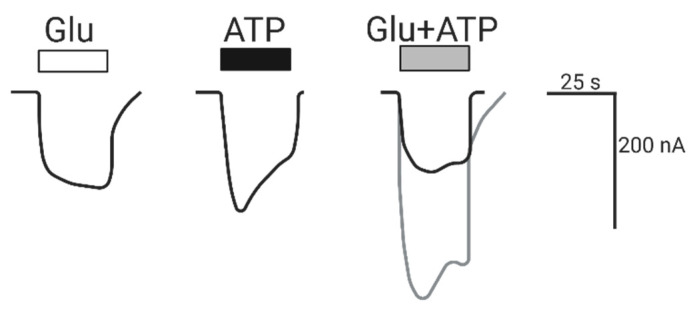
P2X4–NMDAR inhibition is observed at low current responses. Representative current recorded from an individual oocyte expressing P2X4 and GluN2B-containing NMDARs, responding to: Glu (2 µM), ATP (5 µM), or Glu and ATP (2 µM and 5 µM, respectively). The predicted additive response (grey line) is calculated as the sum of the individual Glu and ATP induced currents.

## Abbreviations

P2X	Purinergic receptors
NMDARs	N-methyl-d-aspartate receptors
GABA	γ-aminobutyric acid
AMPA	α-amino-3-hydroxy-5-methyl-4-isoxazolepropionic acid
CT	Carboxy-terminal tail
LGICS	Ligand-gated ion channels
ATP	Adenosine triphosphate
CNS	Central nervous system
P2X4 KO	P2X4 knockout
LTP	Long-term potentiation
LTD	Long-term depression
TEVC	Two-electrode voltage clamp
Glu	Glutamate
CfRS	Calcium-free Ringers’ solution
P2X4-377TR	P2X4 receptor truncated at residue 377
P2X4-382TR	P2X4 receptor truncated at residue 382
P2X4-FlagIN	P2X4 receptor with a FLAG epitope in place of internalization domain (YEQGL)
11C	Peptide consisting of the distal C-terminal 11 amino-acids of P2X4
5-HT_3A_	5-Hydroxytryptamine Receptor 3A

## Appendix A

To evaluate the potential functional interaction between P2X and NMDARs using two-electrode voltage-clamp (TEVC) we coexpressed both receptor types in *Xenopus laevis* oocytes. We performed mRNA injection of P2X or NMDARs at previously reported concentrations, titrating injections until each receptor system produced comparable currents. We then generated eight-point concentration response curves for oocytes expressing either P2X4 or NMDARs (GluN1 and GluN2A or GluN2B or GluN2C). The EC_50_ values for P2X4 and NMDA receptors calculated from ATP and Glu concentration response curves (Figure A1a–d, solid lines), were consistent with previously reported values [38,43]. We then generated an eight-point ATP or Glu concentration response curve for oocytes coexpressing both P2Xs and NMDARs. There were no shifts in concentration response curves when P2X4s and NMDARs were coexpressed (Figure 1a–d, dotted lines vs. solid lines). Consistently, there were no statistically significant differences in the EC50 values for receptors regardless those were expressing individually or in combination (Figure A1). These studies demonstrate that coexpressing both P2X and NMDARs does not change the function of either receptor.
